# Journal Announcements

**Published:** 1911-11

**Authors:** 


### Journal Announcements

PROGRESS.—Eight pages are added to the JOURNAL, beginning with the present issue, mainly to accommodate new advertisements and miscellany. The Original and Abstract Departments will be enlarged soon. The circulation has been increased about 350. Excepting on account of death, there have been only two discontinuances, one by a doctor of over fifty years' practice, who has retired, the other by one who discontinued paying for his JOURNAL some years ago. We appreciate very much the promptness with which subscribers have remitted and the friendly interest manifested in contributing articles, securing new subscribers and in various other ways. Just a word as to the change of staff, to afford local centers for securing news notes, original articles, etc. In welcoming these Associates, we wish to avoid the impression of having used a new broom too vigorously. With one exception, the members of the previous staff who have resigned, had not been in active service for some time, owing to retirement from practice, removal from Buffalo and pressure of practice. For the last reason, the one exception is accounted for but we have the promise of an occasional article from his forcible pen and, indeed, the other members of the previous staff have all promised their support in one way or another.

PREMIUMS.—The BUFFALO MEDICAL JOURNAL does not offer premiums of any kind, mainly for the reason that, if they are of any value, a discrimination is made against old subscribers. We shall be glad, at any time, to afford to any subscriber, whatever advantage belong to the premium system, in regard to securing books, office supplies, etc. Back numbers of the JOURNAL will be supplied free to subscribers, to complete files. We have a few copies of the Transactions of the Medical Society of the State of New York and a set of Foster's Encyclopædic Medical Dictionary for which a nominal price will be charged.

EXPECTED ARTICLES.—Beside the articles previously announced, we expect to publish, during the winter, one from Dr. Louis Faugere Bishop of New York, and one from Sir William Osler, Regius Professor of Medicine, Oxford, England, as well as a number of interesting papers from men in our own territory. Dr. Osler writes under date of September 18, "Dear Benedict:

I am very glad indeed to hear that you are in charge of the BUFFALO MEDICAL JOURNAL, for which I have always had an affection, a good deal, I must say, from the appreciation I had of the splendid men who were its founders and it is a journal, too, that has, all along, done good work."

